# Regioselective synthesis of chiral dimethyl-bis(ethylenedithio)tetrathiafulvalene sulfones

**DOI:** 10.3762/bjoc.11.124

**Published:** 2015-07-02

**Authors:** Flavia Pop, Narcis Avarvari

**Affiliations:** 1 Université d'Angers, CNRS, Laboratoire MOLTECH-Anjou, UMR 6200, UFR Sciences, Bât. K, 2 Bd. Lavoisier, 49045 Angers, France

**Keywords:** chirality, crystal structures, molecular materials, sulfones, tetrathiafulvalenes

## Abstract

Enantiopure (*R*,*R*) and (*S*,*S*)-dimethyl-bis(ethylenedithio)tetrathiafulvalene monosulfones have been synthesized by the aerial oxidation of the chiral dithiolates generated from the propionitrile-protected precursors. Both enantiomers crystallize in the orthorhombic chiral space group *P*2_1_2_1_2_1_. They show a boat-type conformation of the TTF moiety, a rather rigid dithiin sulfone ring and the methyl groups in a bisequatorial conformation. Cyclic voltammetry measurements indicate fully reversible oxidation in radical cation and dication species.

## Introduction

**Scheme 1:**
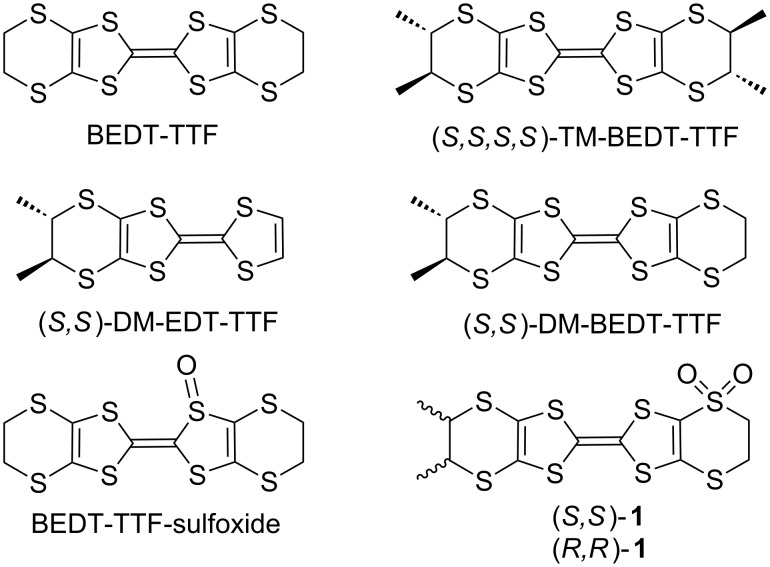
BEDT-TTF and chiral derivatives.

Chiral tetrathiafulvalene (TTF) derivatives have been addressed for the first time in the middle of 80s by Dunitz and Wallis through the synthesis of the (*S*,*S*,*S*,*S*)-enantiomer of tetramethyl-bis(ethylenedithio)tetrathiafulvalene (TM-BEDT-TTF) (Scheme 1) [1], thus opening opportunities towards the preparation of chiral molecular conductors [2]. Since then a large number of chiral TTF derivatives have been prepared [3], especially those derived from bis(ethylenedithio)tetrathiafulvalene (BEDT-TTF) [4]. Although numerous derivatives have been prepared only ten years ago different transport properties were observed for enantiopure and racemic conducting salts based on ethylenedithiotetrathiafulvalene-oxazoline (EDT-TTF-Ox) donors [5–6], due to a structural disorder effect [7]. Evidence was thus provided, and confirmed later on with a second complete series of conducting salts based on the same donors [8], that the presence of chiral centers can modulate the structural disorder of radical cation salts in the solid state, and subsequently, differences in their conducting properties can occur. A similar effect was observed more recently in the [TM-BEDT-TTF][I_3_] family of enantiopure and racemic semiconducting salts [9]. In all these examples the crystal-cell parameters were similar for the enantiopure and racemic salts excepting the space groups which were non-centrosymmetric and centrosymmetric, respectively. On the other hand, complete different solid-state packings may be observed in enantiopure and racemic forms of the same donor, as recently described for a series of mixed valence salts based on the dimethyl-ethylenedithiotetrathiafulvalene (DM-EDT-TTF) precursor (Scheme 1). Here the racemic salt was found to be metallic, while the enantiopure forms showed semiconducting behavior [10]. One of the most important results is the observation of a synergistic effect between chirality and conductivity in enantiopure mixed-valence metallic salts formulated as [DM-EDT-TTF]_2_[ClO_4_] [11]. This is referred to as the electrical magnetochiral anisotropy (eMChA) effect. This effect, which translates the influence of chirality on the transport properties measured in a parallel magnetic field [12], was previously observed only in bismuth wires and carbon nanotubes [13]. Another interesting research area is the redox modulation of the chiroptical properties described in derivatives such as TTF-allenes [14], TTF-helicenes [15], or TTF-paracyclophanes [16]. Thus, to address the different opportunities offered by the combination of chirality with the TTF motif, a certain number of families of precursors have been reported. They possess various types of chirality, i.e., stereogenic centers, axial, planar, helical chirality, and supramolecular chirality [17–21]. Since methylated BEDT-TTF derivatives such as dimethyl-bis(ethylenedithio)tetrathiafulvalene (DM-BEDT-TTF) [22–24], TM-BEDT-TTF [2,9,25–26] and DM-EDT-TTF [10] proved to be the most promising precursors for the preparation of chiral conductors, we were interested in the synthesis of functional derivatives thereof. One of the possibilities hardly addressed so far in TTF chemistry is the oxidation of the sulfur atoms into sulfoxides or sulfones. Indeed, only two reports deal with the oxidation of BEDT-TTF into BEDT-TTF monosulfoxides (Scheme 1), along with enantioselectivity issues [27–28]. We describe herein the synthesis, characterization and solid-state structures of the (*S*,*S*) and (*R*,*R*) enantiomers of DM-BEDT-TTF monosulfones **1** (Scheme 1).

## Results and Discussion

In our previous studies dealing with the sulfoxidation of the BEDT-TTF donor we could selectively obtain the *inner* BEDT-TTF sulfoxide (Scheme 1) by using chiral sulfonyl-oxaziridines as oxidizing agent [27–28]. However, the *inner* BEDT-TTF sulfoxide was shown to be of only limited interest as precursor for molecular conductors, since it does not reversibly oxidize into a radical cation. This behavior is due to the moderate kinetic stability of the latter, which releases oxygen to transform into BEDT-TTF. Moreover, since the *inner* sulfur atoms present large orbital coefficients in the HOMO, the introduction of the electron-withdrawing oxygen atom induces a massive increase of the oxidation potential from the neutral to the radical cation states. We have then hypothesized that the oxidation of the *outer* sulfur atoms into sulfoxide or sulfone should only moderately influence the oxidation potential and thus provide more stable radical cation species. In order to access chiral precursors with controlled stereochemistry we decided to investigate the sulfoxidation of the DM-BEDT-TTF precursor.

**Scheme 2:**
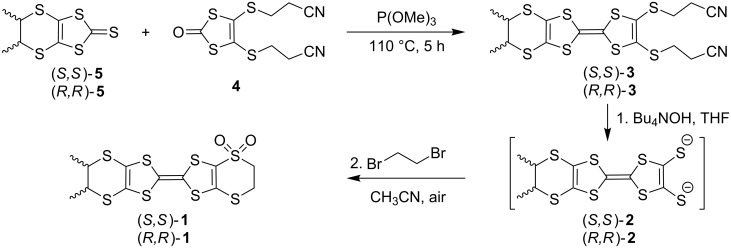
Synthesis of the chiral sulfones (*S,S*)-**1** and (*R,R*)-**1**.

Compounds (*S*,*S*)-**1** and (*R*,*R*)-**1** were synthesized in two steps from the corresponding enantiopure dithiole-thiones (*S*,*S*)-**5** and (*R*,*R*)-**5** and the dithiolone-dithiopropionitrile **4** (Scheme 2). In the first step the phosphite-mediated heterocoupling between the two units provides the enantiomeric DM-EDT-TTF-dithiopropionitriles (*S*,*S*)-**3** and (*R*,*R*)-**3** as the major products. Tetrabutylammonium hydroxide was then used to generate the corresponding dithiolates **2** in THF that, after solvent evaporation during which air was allowed in the Schlenk tube, were further refluxed in acetonitrile with 1,2-dibromoethane to afford the chiral monosulfones (*S*,*S*)-**1** and (*R*,*R*)-**1**. Thus, the oxidation of one of the outer sulfur atoms takes place in situ as the intermediate DM-EDT-TTF dithiolates are reactive towards oxygen. Interestingly, the reaction is regio- and chemoselective, as only compound **1** was isolated after column chromatography.

We have thus succeeded, through this simple strategy, to selectively oxidize one of the *outer* sulfur atoms of the DM-EDT-TTF donor, which represents a remarkable regio- and chemoselectivity.

**Figure 1:**
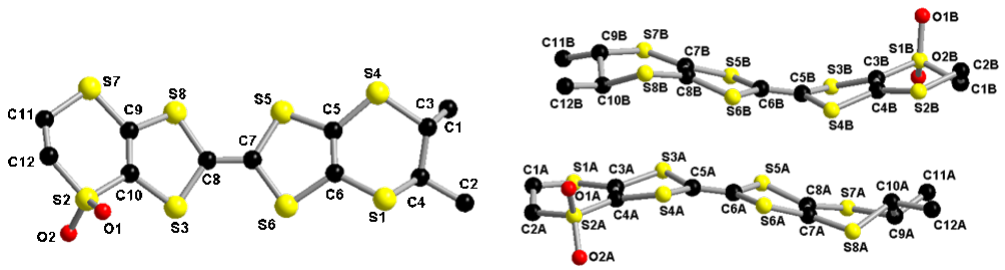
Molecular structure of (*R*,*R*)-**1** (left) and (*S*,*S*)-**1** (right) together with atom numbering scheme (H atoms have been omitted for clarity).

Besides NMR (Figures S1 and S2 in Supporting Information File 1), mass spectrometry and elemental analysis, which are all concordant, the definite proof for the sulfone structure of **1** has been provided by single crystal X-ray diffraction analysis. Single crystals of the two enantiomers (*S*,*S*)-**1** and (*R*,*R*)-**1** were obtained by slow evaporation from dichloromethane/pentane or dichloromethane solutions, respectively. Although both enantiomers crystallize in the orthorhombic system (chiral space group *P*2_1_2_1_2_1_), they are not isostructural, very likely because of the slightly different crystallization conditions. Indeed, the cell parameters are completely different and in the asymmetric unit of (*R*,*R*)-**1** there is one molecule and in that of (*S*,*S*)-**1** there are two independent molecules (Figure 1).

**Figure 2:**
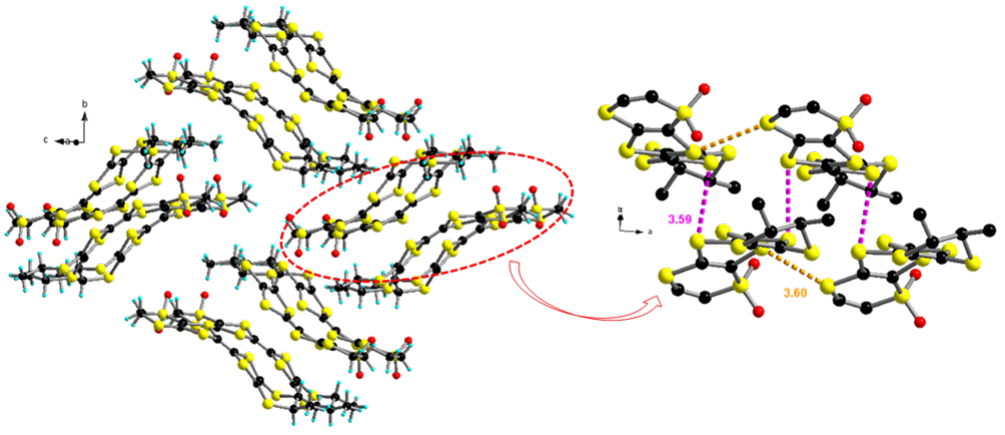
Packing of (*R*,*R*)-**1** in the *bc* plane (left) and detailed S···S interactions (only S3···S7 (−1+x, y, z) 3.60 Å and S6···S8 (−0.5+x, 0.5−y, 1−z) of 3.59 Å are highlighted) between molecules in each stack (right).

In both structures the methyl groups adopt equatorial positions, unlike the structure of DM-BEDT-TTF, in which they are axially oriented [22]. We have previously shown that for the TM-BEDT-TTF donor the axial orientation is slightly more favored in the gas phase than the equatorial one, but both can occur in the solid state [9,25]. In some cases even mixed (ax,ax,eq,eq) conformations have been observed in the solid state [25–26,29]. In the (*R*,*R*)-**1** molecule the dithiole and the methyl-substituted dihydrodithiin rings show rather strong distortions, with dihedral angles about the S···S hinges of 27.6° (S3–S8), 22.1° (S5–S6) and 46.1° (S1–S4). On the contrary, the dihydrodithiin sulfone ring is almost planar, with a S2···S7 folding angle of only 5.3°, certainly because of the rigidity imposed by the tetrahedral R_2_SO_2_ sulfur atom. The S=O bond lengths have values of 1.396(5) Å for S2=O1 and 1.380(5) Å for S2=O2, which are somewhat shorter than the usual values of 1.43–1.44 Å reported in the literature [30–32] (Table 1). Moreover, these S=O bonds are shorter when compared to those in *inner* TTF sulfoxides [27–28], yet the same feature was already observed in other sulfoxide/sulfone series [30–31]. The central C7=C8 bond measures 1.346(6) Å, which is a typical value for a neutral donor. In the packing the donors interact laterally along the *a* direction, with the shortest intermolecular S···S distance of 3.60 Å for S3···S7 (−1+x, y, z), forming chains which further dimerize through S6···S5 (−0.5+x, 0.5−y, 1−z) and S6···S8 (−0.5+x, 0.5−y, 1−z) contacts amounting to 3.54 and 3.59 Å, respectively. Then, the dimerized chains arrange perpendicular to each other in the *bc* plane (Figure 2).

**Table 1:** Selected bond distances for (*R*,*R*)-**1** and (*S*,*S*)-**1**.

Bond lengths (Å)		

(*R*,*R*)-**1**	(*S*,*S*)-**1 A**	(*S*,*S*)-**1 B**

C7=C8···1.346(6)	C5=C6···1.347(7)	C5=C6···1.347(7)
S5–C7···1.759(5)	S3–C5···1.770(5)	S3–C5···1.760(5)
S6–C7···1.748(5)	S4–C5···1.751(6)	S4–C5···1.755(5)
S8–C8···1.762(6)	S5–C6···1.741(6)	S5–C6···1.751(5)
S3–C8···1.771(6)	S6–C6···1.766(5)	S6–C6···1.750(5)
S2–O1···1.396(5)	S2–O1···1.395(5)	S1–O1···1.435(5)
S2–O2···1.380(5)	S2–O2···1.404(5)	S1–O2···1.430(4)

**Figure 3:**
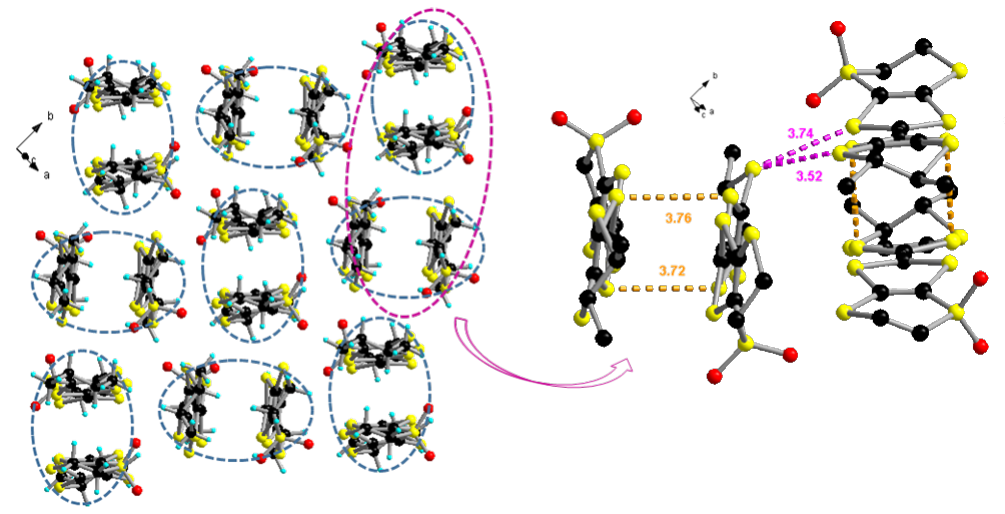
Packing of (*S*,*S*)-**1** in the *ab* plane (left) and detailed S···S intermolecular interactions within (highlighted S5A···S5B (2−x, −0.5+y, 0.5−z) 3.72 Å and S6A···S6B (2−x, −0.5+y, 0.5−z) 3.72 Å) and between (highlighted S5B···S8B (2−x, 0.5+y, 0.5−z) 3.72 Å and S3B···S8B (2−x, 0.5+y, 0.5−z) 3.74 Å) orthogonal dimers (right).

In the structure of (*S*,*S*)-**1** the overall configuration of the two independent molecules is similar, of boat type, with distortions along the internal S···S axes in the same sense, having rather close values of 23.5° (S3A···S4A) and 16.7° (S5A···S6A) for molecule A, and 27.4° (S3B···S4B) and 14.4° (S5B···S6B) for molecule B. As far as the dihydrodithiin rings are concerned, the methyl-substituted ones are much less folded, according to the dihedral angles of 8.8° (S1A···S2A) and 16.5° (S1B···S2B), compared to those in the unsubstituted rings, amounting to 26.9° (S7A···S8A) and 22.1° (S7B···S8B). The S=O-bond lengths range between 1.395(5) and 1.435(5) Å, while the central C5=C6 bonds have the same value of 1.347(7) Å for both A and B molecules, typical for neutral donors (Table 1). (*S*,*S*)-**1** packs in *pseudo*-centrosymmetric head-to-tail dimers which are orthogonally disposed in an edge-to-face arrangement (Figure 3). The shortest intermolecular S···S distances within the dimers are 3.72 Å for S5A···S5B (2−x, −0.5+y, 0.5−z) and 3.76 Å for S6A···S5B (2−x, −0.5+y, 0.5−z), while they are smaller between dimers, as for example S5B···S8B (2−x, 0.5+y, 0.5−z) (3.52 Å) or S6A···S7A (3−x, −0.5+y, 0.5−z) (3.49 Å).

A very important aspect related to the interest of these chiral TTF sulfones as precursors for molecular conductors is the stability of the radical cation species. As mentioned earlier, the major drawback of the *inner* BEDT-TTF sulfoxides is their irreversible oxidation, as the radical cations once generated lose oxygen to afford BEDT-TTF. However, in strike contrast with the behavior of the latter, cyclic voltammetry measurements for (*R*,*R*)-**1** and (*S*,*S*)-**1** show reversible two single-electron oxidation processes into radical cation and dication species at Δ*E*_1/2_ = +0.67 and 1.00 V vs SCE, respectively (Figure S3 in Supporting Information File 1). The first value is thus largely cathodically shifted with respect to the oxidation of the *inner* BEDT-TTF sulfoxide occurring at +0.95 V vs SCE, and only slightly anodically shifted when compared to the DM-BEDT-TTF donor [22].

## Conclusion

Enantiopure (*R*,*R*) and (*S*,*S*)-dimethyl-bis(ethylenedithio)tetrathiafulvalene (DM-BEDT-TTF) monosulfones have been selectively prepared by the in situ aerial oxidation of a TTF dithiolate precursor followed by quenching with dibromoethane. Both enantiomers have been thoroughly characterized in solution and in the solid state by single crystal X-ray diffraction. The conformation of the enantiomers is very similar in the solid state, including the equatorial position of the methyl substituents, in spite of their different packing diagrams which are dominated by the intermolecular S···S interactions. The electrochemical behavior of these *outer* DM-BEDT-TTF sulfones is strikingly different from the one of the *inner* BEDT-TTF sulfoxide, as it shows fully reversible oxidation processes at much lower potentials. Accordingly, these new donors represent valuable precursors for crystalline chiral radical cation salts. Moreover, the partial reduction to the corresponding *outer* sulfoxides, which would generate an additional stereogenic center at the SO sulfur atom can be envisaged. These aspects are currently addressed in our laboratory.

## Experimental

**Materials and instrumentation:** Reactions were carried out under argon; dry solvents were obtained from solvent drying machines. Nuclear magnetic resonance spectra were recorded on Bruker Avance DRX 300 and 500 spectrometers operating at 300 and 500 MHz for ^1^H and 75 and 125 MHz for ^13^C, respectively. Chemical shifts are expressed in parts per million (ppm) downfield from external TMS. The following abbreviations are used: d, doublet; t, triplet; m, multiplet. MALDI–TOF MS spectra were recorded on a Bruker Biflex-IIITM apparatus, equipped with a 337 nm N_2_ laser. Elemental analyses were recorded using Flash 2000 Fisher Scientific Thermo Electron analyzer. The starting compounds **4** [33] and **5** [10] have been prepared as described in the literature.

### Syntheses

**(*S*,*S*)-3**: A mixture of (*S*,*S*)-**5** (0.56 g, 2.21 mmol) and 3,3'-((2-oxo-1,3-dithiole-4,5-diyl)bis(sulfanediyl))dipropanenitrile (**4**, 1.23 g, 4.36 mmol, 2 equiv) in 10 mL of freshly distilled trimethylphosphite was heated under argon at 110 °C for 5 h. The solvent was evaporated in a rotary evaporator, and then 20 mL of toluene were added and evaporated. The last procedure was repeated twice. The product was dissolved in dichloromethane and passed through a silica column to remove the remaining phosphate, and then purified by chromatography using petroleum spirit/dichloromethane 1:1 followed by dichloromethane as eluent, to afford an orange solid (0.53 g, 48%). ^1^H NMR (300 MHz, CDCl_3_) δ 3.23–3.17 (m, 2H, -C*H*-CH_3_), 3.06 (t, 2H, -C*H_2_-*), 2.71 (t, 2H, -C*H_2_-*), 1.42 (d, 6H, -C*H_3_*) ppm; MALDI–TOF MS (*m*/*z*): [M − CH_2_CH_2_CN)]^+^ 437.4; Anal. calcd for C_16_H_16_N_2_S_8_: C, 38.99; H, 3.27; N, 5.68; S, 52.05; found: C, 38.65; H, 3.05; N, 5.34; S, 52.43 (%).

**(*R*,*R*)-3**: The same synthetic procedure was followed as for the (*S,S*) enantiomer starting from (*R,R*)-**5**. Yield 55%. Anal. calcd for C_16_H_16_N_2_S_8_: C, 38.99; H, 3.27; N, 5.68; S, 52.05; found: C, 38.71; H, 3.08; N, 5.32; S, 52.51 (%).

**DM-BEDT-TTF monosulfone (*S*,*S*)-1:** After the solution of (*S*,*S*)-**3** (100 mg, 0.2 mmol) in 7 mL of dry THF was degassed for 10 min by bubbling argon through the solution, tetrabutylammonium hydroxide solution (1 M in methanol, 0.44 mL, 0.44 mmol) was added and the mixture was stirred at rt for 30 min. Then the THF was evaporated under vacuum, 10 mL of dry acetonitrile were added and the mixture was refluxed for 2 h. After the solution was concentrated the crude reaction mixture was chromatographed on silica gel using dichloromethane/pentane 1:1 to up to 4:1 as eluent to afford a yellow orange solid (27 mg, 30%). Single crystals were obtained by slow evaporation of a dichloromethane solution. ^1^H NMR (500 MHz, CD_2_Cl_2_) δ 3.71–3.68 (m, 2H, -SC*H_2_*-), 3.56–3.53 (m, 2H, -C*H_2_*-S), 3.27–3.21 (m, 2H, S-C*H*-C*H_3_*), 1.42 (d, 6H, -C*H_3_*) ppm; ^13^C NMR (100 MHz, CD_2_Cl_2_) δ 136.06, 120.59, 116.56, 112.02, 111.44, 50.10, 44.04, 30.38, 27.39, 21.36 ppm; MALDI–TOF MS (*m*/*z*): 444 [M]^+^ (M_calcd_ = 443.86); Anal. calcd for C_12_H_12_O_2_S_8_: C, 32.41; H, 2.72; O, 7.19; S, 57.68; found: C, 32.72; H, 2.55; O, 6.95; S, 57.93 (%).

**(*R*,*R*)-1**: The same synthetic procedure was followed as for the (*S,S*)-enantiomer starting from (*R*,*R*)-**3**. Yellow orange solid (32 mg, 35%). Single crystals were obtained by slow evaporation of a dichloromethane solution. Anal. calcd for C_12_H_12_O_2_S_8_: C, 32.41; H, 2.72; O, 7.19; S, 57.68; found: C, 32.68; H, 2.61; O, 7.01; S, 57.88 (%).

**Crystallography:** X-ray diffraction measurements were performed on a Nonius Kappa CCD diffractometer, using graphite-monochromated Mo Kα radiation (λ = 0.71073 Å). The structures were solved (SHELXS-97) by direct methods and refined (SHELXL-97) by full-matrix least-square procedures on F^2^ [34]. The non-H atoms were refined with anisotropic displacement parameters. A summary of the crystallographic data and the structure refinement is given in Table 2. CCDC reference numbers: CCDC 1057825 (*R*,*R*)-**1** and CCDC 1057826 (*S*,*S*)-**1**. These data can be obtained free of charge from The Cambridge Crystallographic Data Centre via http://www.ccdc.cam.ac.uk/data_request/cif.

**Table 2:** Crystallographic data, details of data collection and structure refinement parameters for (*S*,*S*)-**1** and (*R*,*R*)-**1**.

	(*R*,*R*)-**1**	(*S*,*S*)-**1**

Moiety formula	C_12_H_12_O_2_S_8_	C_12_H_12_O_2_S_8_
*M* [gmol^−1^]	444.70	444.70
*T* [K]	293(2)	293(2)
Crystal system	Orthorhombic	Orthorhombic
Space group	P*212121*	P*212121*
*a* [Å]	6.9459(9)	10.1062(9)
*b* [Å]	15.258(2)	11.5753(12)
*c* [Å]	16.523(3)	29.583(2)
α [°]	90.000	90.000
β [°]	90.000	90.000
γ [°]	90.000	90.000
*V* [Å^3^]	1751.1(4)	3460.7(6)
*Z*	4	8
ρ*_calcd_* [g cm^−3^]	1.687	1.707
*μ* [mm^−1^]	1.020	1.032
Goodness-of-fit on F^2^	1.077	1.040
Final R1/*w*R2 [I > 2σ(I)]	0.0429/0.0809	0.0591/0.0978
R1/*w*R2 (all data)	0.0737/0.0910	0.1058/0.1089

**Electrochemical studies**: Cyclic voltammetry measurements were carried out with a Biologic SP-150 potentiostat in a glove box containing dry, oxygen-free (<1 ppm) argon at 293 K, by using a three-electrode cell equipped with a platinum millielectrode of 0.126 cm^2^ area, an Ag/Ag^+^ pseudo-reference electrode and a platinum-wire counter electrode. The potential values were then re-adjusted with respect to the saturated calomel electrode (SCE). The electrolytic media involved a 0.1 mol/L solution of (*n*-Bu_4_N)PF_6_ in CH_2_Cl_2_/acetonitrile 1:1. All experiments were performed at room temperature at 0.1 V/s.

## Supporting Information

File 1 ^1^H NMR spectra of (*S*,*S*)-**3** and (*S*,*S*)-**1** and cyclic voltammogram of (*S*,*S*)-**1**.

## References

[1] Wallis J D, Karrer A, Dunitz J D (1986). Helv Chim Acta.

[2] Karrer A, Wallis J D, Dunitz J D, Hilti B, Mayer C W, Bürkle M, Pfeiffer J (1987). Helv Chim Acta.

[3] Avarvari N, Wallis J D (2009). J Mater Chem.

[4] Griffiths J-P, Nie H, Brown R J, Day P, Wallis J D (2005). Org Biomol Chem.

[5] Réthoré C, Fourmigué M, Avarvari N (2004). Chem Commun.

[6] Réthoré C, Fourmigué M, Avarvari N (2005). Tetrahedron.

[7] Réthoré C, Avarvari N, Canadell E, Auban-Senzier P, Fourmigué M (2005). J Am Chem Soc.

[8] Madalan A M, Réthoré C, Fourmigué M, Canadell E, Lopes E B, Almeida M, Auban-Senzier P, Avarvari N (2010). Chem – Eur J.

[9] Pop F, Laroussi S, Cauchy T, Gómez-García C J, Wallis J D, Avarvari N (2013). Chirality.

[10] Pop F, Auban-Senzier P, Frąckowiak A, Ptaszyński K, Olejniczak I, Wallis J D, Canadell E, Avarvari N (2013). J Am Chem Soc.

[11] Pop F, Auban-Senzier P, Canadell E, Rikken G L J A, Avarvari N (2014). Nat Commun.

[12] Rikken G L J A, Fölling J, Wyder P (2001). Phys Rev Lett.

[13] Krstić V, Roth S, Burghard M, Kern K, Rikken G L J A (2002). J Chem Phys.

[14] Hasegawa M, Sone Y, Iwata S, Matsuzawa H, Mazaki Y (2011). Org Lett.

[15] Biet T, Fihey A, Cauchy T, Vanthuyne N, Roussel C, Crassous J, Avarvari N (2013). Chem – Eur J.

[16] Kobayakawa K, Hasegawa M, Sasaki H, Endo J, Matsuzawa H, Sako K, Yoshida J, Mazaki Y (2014). Chem – Asian J.

[17] Saad A, Jeannin O, Fourmigué M (2011). Tetrahedron.

[18] Danila I, Riobé F, Piron F, Puigmartí-Luis J, Wallis J D, Linares M, Ågren H, Beljonne D, Amabilino D B, Avarvari N (2011). J Am Chem Soc.

[19] Danila I, Pop F, Escudero C, Feldborg L N, Puigmartí-Luis J, Riobé F, Avarvari N, Amabilino D B (2012). Chem Commun.

[20] Awheda I, Krivickas S J, Yang S, Martin L, Guziak M A, Brooks A C, Pelletier F, Le Kerneau M, Day P, Horton P N (2013). Tetrahedron.

[21] Pop F, Melan C, Danila I, Linares M, Beljonne D, Amabilino D B, Avarvari N (2014). Chem – Eur J.

[22] Matsumiya S, Izuoka A, Sugawara T, Taruishi T, Kawada Y (1993). Bull Chem Soc Jpn.

[23] Matsumiya S, Izuoka A, Sugawara T, Taruishi T, Kawada Y, Tokumoto M (1993). Bull Chem Soc Jpn.

[24] Pop F, Allain M, Auban-Senzier P, Martínez-Lillo J, Lloret F, Julve M, Canadell E, Avarvari N (2014). Eur J Inorg Chem.

[25] Yang S, Pop F, Melan C, Brooks A C, Martin L, Horton P, Auban-Senzier P, Rikken G L J A, Avarvari N, Wallis J D (2014). CrystEngComm.

[26] Atzori M, Pop F, Auban-Senzier P, Clérac R, Canadell E, Mercuri M L, Avarvari N (2015). Inorg Chem.

[27] Chas M, Lemarié M, Gulea M, Avarvari N (2008). Chem Commun.

[28] Chas M, Riobé F, Sancho R, Minguíllon C, Avarvari N (2009). Chirality.

[29] Pop F, Lacour J, Avarvari N (2012). Rev Roum Chim.

[30] Wieland T, Götzendörfer C, Dabrowski J, Lipscomb W N, Shoham G (1983). Biochemistry.

[31] Bouamaied I, Constable E C, Housecroft C E, Neuburger M, Zampese J A (2012). Dalton Trans.

[32] de León A, Guerrero M, García-Antón J, Ros J, Font-Bardía M, Pons J (2013). CrystEngComm.

[33] Svenstrup N, Rasmussen K M, Hansen T K, Becher J (1994). Synthesis.

[34] (1996). Programs for the Refinement of Crystal Structures.

