# Supplementary material for: Regioselective synthesis of chiral dimethyl-bis(ethylenedithio)tetrathiafulvalene sulfones
Source: Beilstein J Org Chem. 2015 Jul 2;11:1105–11. doi: 10.3762/bjoc.11.124 (PMC4505188; doi:10.3762/bjoc.11.124)
Supplement: File 1 — 1H NMR spectra of (S,S)-3 and (S,S)-1 and cyclic voltammogram of (S,S)-1. [file Beilstein_J_Org_Chem-11-1105-s001.pdf]

# Supporting Information

## for

# Regioselective synthesis of chiral dimethyl-bis(ethylenedithio)tetrathia-fulvalene sulfones

Flavia Pop and Narcis Avarvari*

Address: Université d'Angers, CNRS, Laboratoire MOLTECH-Anjou, UMR 6200, UFR Sciences, Bât. K, 2 Bd. Lavoisier, 49045 Angers, France.

Email: Narcis Avaravari - narcis.avarvari@univ-angers.fr

*Corresponding author

## NMR spectra and cyclic voltammograms

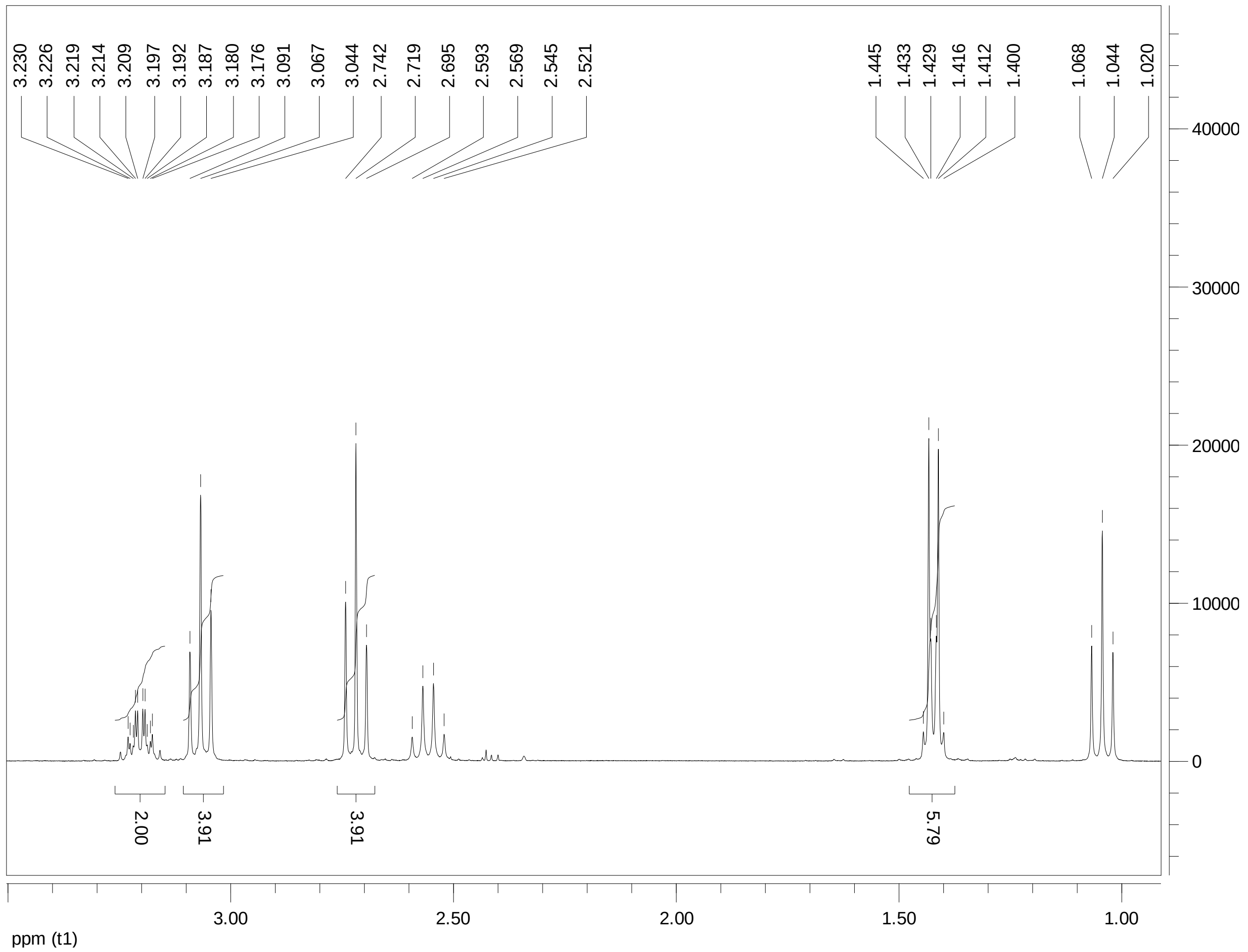


**Figure S1:** $^1$H NMR spectrum of (*S*,*S*)-**3**, in $CDCl_3$ with triethylamine.

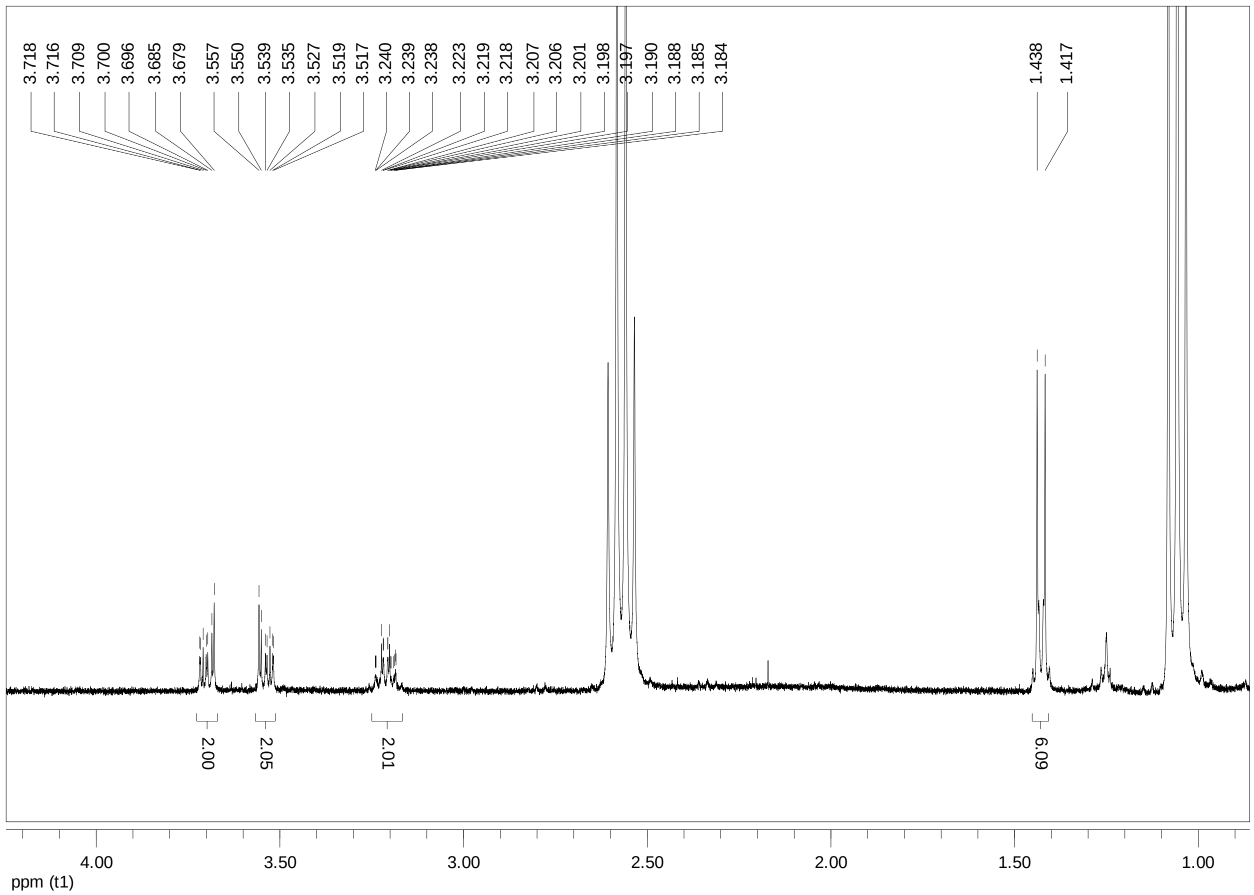


**Figure S2:** $^{1}$H NMR spectrum of (*S*,*S*)-**1** in $CDCl_3$ with triethylamine.

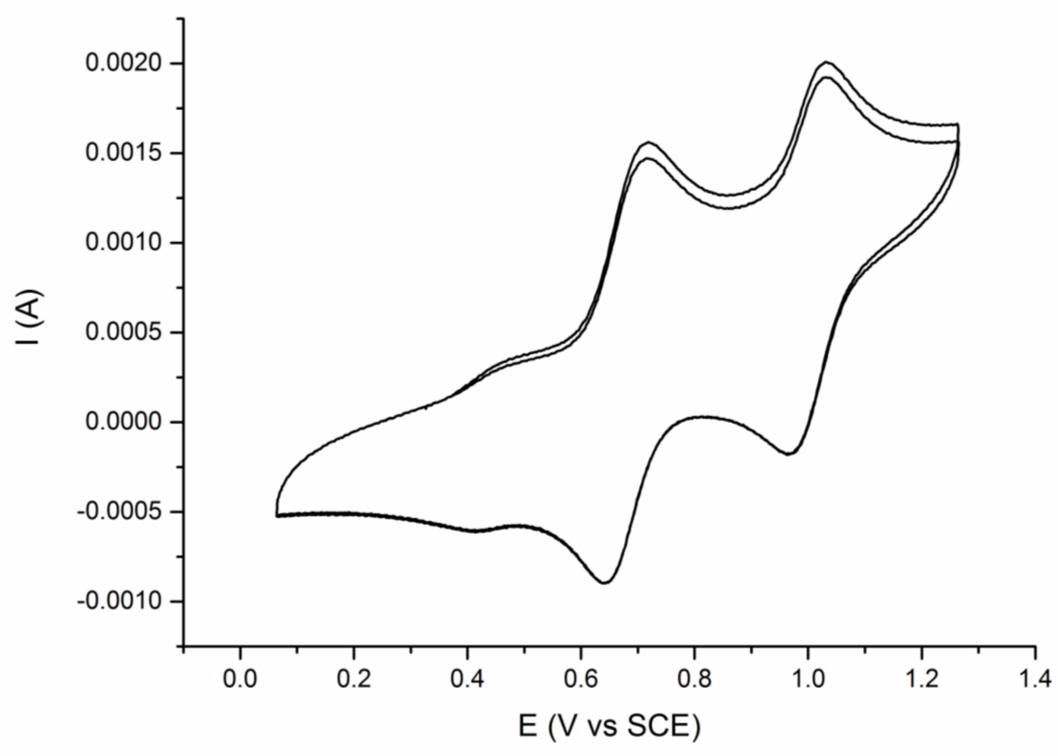


**Figure S3:** Cyclic voltammogram of (*S*,*S*)-**1** in $10^{-4}$ M in DCM/ACN 1:1. $E^{1/2}_{ox1}$ = 0.67 V; $E^{1/2}_{ox2}$= 1 V vs SCE.
